# Allelic Imbalance Analysis in Liquid Biopsy to Monitor Locally Advanced Esophageal Cancer Patients During Treatment

**DOI:** 10.3389/fonc.2020.01320

**Published:** 2020-08-28

**Authors:** Elisa Boldrin, Matteo Curtarello, Matteo Fassan, Massimo Rugge, Stefano Realdon, Rita Alfieri, Alberto Amadori, Daniela Saggioro

**Affiliations:** ^1^Immunology and Molecular Oncology, Veneto Institute of Oncology IOV-IRCCS, Padova, Italy; ^2^Department of Medicine (DIMED), Surgical Pathology and Cytopathology, University of Padova, Padova, Italy; ^3^Endoscopy Unit, Veneto Institute of Oncology IOV-IRCCS, Padova, Italy; ^4^Oncological Surgery, Veneto Institute of Oncology IOV-IRCCS, Padova, Italy; ^5^Department of Surgical Sciences, Oncology and Gastroenterology, University of Padova, Padova, Italy

**Keywords:** esophageal cancer, esophageal adenocarcinoma (EADC), esophageal squamous cell carcinoma (ESCC), circulating cell free DNA (cfDNA), liquid biopsy, longitudinal studies, loss of heterozygosis (LOH), allelic imbalance

## Abstract

Esophageal cancer (EC) is a highly aggressive tumor, and the current monitoring procedures are partially inadequate to evaluate treatment efficacy. The aim of this study was to investigate whether allelic imbalance analysis in liquid biopsy could be used as an additional tool to monitor tumor burden in EC patients. For this purpose, circulating cell-free DNA (cfDNA) from 52 patients with a locally advanced EC, which underwent neoadjuvant treatment and resection, was analyzed. Data from four representative longitudinally followed patients are also reported. Furthermore, 17 DNAs from formalin-fixed paraffin-embedded (FFPE) tumor samples were analyzed and compared to time-matched cfDNAs. To look for allelic imbalance, which is the main genetic alteration in both EC histotypes, we used a panel of five microsatellites (MSs) and three single-nucleotide polymorphisms (SNPs) near genes described as frequently altered. The Fisher exact and Mann-Whitney *U* tests were used to analyze categorical and continuous data, respectively. The correlation coefficient between cfDNA and FFPE-DNA was calculated with the Pearson's correlation test. We found that the selected tumor-related alterations are present in cfDNA of both adenocarcinoma (EADC) and squamous cell carcinoma (ESCC) with similar frequencies. The only exception were the MSs, one downstream and one upstream, of *SMAD4* of which the loss was only observed in EADC (26 vs. 0%, *P* = 0.018). More interestingly, longitudinal studies disclosed that in patients with disease progression, tumor-related alterations were present in cfDNA before overt clinical or instrumental signs of relapse. In conclusion, our data indicate that the evaluation of tumor-related gene allelic imbalance in cfDNA might be a useful tool to complement the current monitoring procedures for EC patients and to guide their management.

## Introduction

Esophageal cancer (EC) is a highly aggressive tumor, and the majority of patients die of recurrent disease within 2 years from diagnosis; this happens even after a putative radical esophagectomy (1, 2). EC presents two main histotypes, which occur in distinct esophageal districts: adenocarcinoma (EADC) and squamous cell carcinoma (ESCC). EADC develops in the lower esophagus, near the gastroesophageal junction, while ESCC arises in the mid upper esophagus. The incidence of EADC is rising in the Western countries where it now represents the most diffuse histotype; ESCC instead remains the most common EC throughout Asia (1).

From the genetic point of view, both EC subtypes are mainly dominated by allelic imbalance, with frequent tumor suppressor gene mutations and losses and oncogene amplifications, as reported in the Cancer Genome Atlas study (3). Moreover, EADC and ESCC show differences at the molecular level. EADC is more similar to the chromosomal unstable (CIN) gastric adenocarcinoma subtype, while ESCC is molecularly closer to head and neck tumors (4). EADCs present frequent amplifications of *ERBB2* (32%), *VEGFA* (28%), *GATA6* (21%) and *GATA4* (21%), and deletion of *SMAD4* (24%), while ESCCs exhibit the prevalent amplification of *CCDN1* (57%), *TP63* (48%), and *EGFR* (19%). Both histotypes have high frequencies of *TP53* (73 vs. 92%) and *CDKN2A* (76 vs. 76%) inactivation and *MYC* amplification (32 vs. 23%) (3, 5).

In recent years, with the increasing knowledge of esophageal tumor genetics, clinical trials of targeted therapy have been conducted, especially using anti-HER2 and anti-VEGF drugs for EADC and anti-EGFR for ESCC; immunotherapy trials are still at an early stage (6–10). However, despite the introduction of these new options, the outcome of EC patients did not improve, and the standard of care for locally advanced tumors remains preoperative treatment with common chemotherapeutic drugs (platinum derivatives, 5-Fluorouracil, taxane, antracyclines), flanked by radiation, and followed by surgery (11, 12). No clear data on the benefit of adjuvant treatment exist (13, 14). For this reason, when a R0 resection is achieved, adjuvant treatment is provided at disease recurrence [i.e., Italian Association of Medical Oncology (AIOM) 2018 guidelines for esophageal cancer treatment].

The poor outcome of EC patients is usually ascribable to diagnosis at an advanced stage, but it is also related to the inadequacy of the current monitoring practices that are not always able to evaluate the effectiveness of treatment or to assess the likelihood of relapse, causing a delay in the administration of efficient therapies.

Recently, circulating cell-free DNA (cfDNA) emerged as a promising tool to diagnose and to monitor tumor behavior in terms of response to treatment, detection of minimal residual disease, and recurrence (15). Moreover, it has been suggested that cfDNA could represent, more efficiently than traditional biopsies, the real status of the tumor by overcoming the challenge of the intra-tumor heterogeneity (16–19).

In this pilot study, we explored the possibility of using liquid biopsy (cfDNA) as a possible additional strategy to follow EC patients during their therapeutic iter. cfDNAs of EC patients, together with DNAs of longitudinally collected samples and time-matched tumor specimens, were analyzed for the presence of tumor-related allelic imbalance events, such as the loss of heterozygosity (LOH) of tumor suppressor genes and the amplification of oncogenes, using a panel of 5 microsatellites (MSs) and 3 single-nucleotide polymorphisms (SNPs). Mutational analysis is currently the most common approach to test the presence of cancer-related alterations in cfDNA. However, we chose allelic imbalance analysis since we believe that this approach allows a more accurate detection of tumor-related alterations, usually present at low frequency in cfDNA. Indeed, LOH that involves the loss of the wild-type allele is needed to unmask somatic mutations occurring in tumor suppressor genes (20). Consequently, at a given locus, the frequency of LOH is generally similar to the frequency of a mutation in a hot-spot region. Conversely, when at a given locus we are faced with different low-frequency mutations, the LOH occurrence is usually higher than that of the single mutation; in this case, the loss of the wild-type allele includes more than one mutation (20).

## Materials and Methods

### Patients

For this pilot study, 52 consecutive EC patients were selected among those who were referred to the Veneto Institute of Oncology IOV-IRCCS between 2013 and 2017. Inclusion criteria consisted of a T3-T4 classification at the time of primary staging or at reevaluation after neoadjuvant chemotherapy, and the availability of clinicopathological data and plasma. Patients with previous neoplasia at other sites or affected by major comorbidities were excluded. Among 10 EC patients for which samples were consecutively collected, four were chosen for longitudinal study on the basis of their clinical history and availability of clinical data. To evaluate the concordance between cfDNA and tumor DNA, 17 time-matched formalin-fixed, paraffin-embedded (FFPE) EADC specimens were also analyzed.

### DNA Extraction

Venous blood samples were collected in EDTA tubes and processed within 2 h. Plasma was isolated from corpuscular components of the blood by centrifugation at 2,000×*g*; plasma was centrifuged a second time at 16,000×*g* to remove cellular debris, and then stored at −80°C until cfDNA extraction. One aliquot of whole blood was also stored for future germline DNA extraction. cfDNA was extracted from 1 ml of plasma using the QIAamp Circulating Nucleic Acid Kit (Qiagen, Milan, Italy); the amount of cfDNA ranged between 500 and 750 ng/ml plasma. Germline DNA was isolated from 250 μl of peripheral blood with the MagNA Pure Compact Nucleic Acid Isolation Kit I using the automated extractor MagNA Pure Compact Instrument (Roche, Milan, Italy), following the manufacturer's recommendations. FFPE tumor DNA was isolated from eight consecutive 10-μm-thick sections using QIAamp Mini Kit (Qiagen, Milan, Italy). A neoplastic component ≥70% was considered adequate for tumor DNA analysis; when necessary, samples were enriched by manual macro-dissection. DNA quantity and quality were assessed with the NanoDrop 1000 spectrophotometer (Thermo Fisher Scientific, Monza, Italy). The cfDNA quality of samples selected at random was further evaluated by means of the Agilent 2100 Bioanalyzer using the High Sensitivity DNA kit (Agilent Technologies, Milan, Italy).

### Genetic Analysis

As a marker of LOH, we used the following MSs: D9S171 in chr. 9p21.3, at 2.5 Mb downstream of *CDKN2A/2B*; D17S796 in chr.17p13.2 and D17S578 in chr.17p13.1 at 1.3 and 0.75 Mb upstream of *TP53*, respectively; D18S363 and D18S474 in chr.18q21.2 at 0.27 Mb upstream and 0.08 Mb downstream of *SMAD4*, respectively. The following SNPs were used as markers of allelic imbalance: rs28673064 located in the 5'UTR of *TP63* (chr.3q28), rs9344 in chr.11q13.3 within the exon 4 of *CCND1*, and rs11078663 in 17p13.1 at 0.63 Mb upstream of *TP53* (Table 1). These markers were selected based on their good frequency of heterozygosity (informativeness) in the Caucasian population (range 56–85%) and chromosomal position proximate to or within tumor suppressor genes and oncogenes known to be frequently altered in EC (3, 21). A small size of the amplification products was another requirement to successfully amplify cfDNA. Primer sequences, obtained from the UCSC Genome Browser (GRCh38/hg38 Assembly), and PCR conditions are reported in Table 1. All selected markers have an appropriate size range to amplify cfDNA fragments, with the exception of rs11078663 and D18S363 that have a suboptimal size (Table 1). However, we decided to include also these two markers since they map in regions covered by other more appropriate markers, making us more confident about the results. For PCR amplification, we used 20–30 ng of DNA.

**Table 1:** Markers used in genetic analysis.

**Microsatellite or SNP**	**Heterozygosity (%)**	**Gene**	**Distance from gene**	**PCR Conditions**	**Size**	**Primers**
rs28673064 (3q28)	53	*TP63*	5′UTR	35 cycles: 95°C 1 min, 56°C 1 min, 72°C 1 min; final extension: 72°C 45 min	180 bp	Fw: TGAAGGAGAGAAGTGCCTAAAC
						Rw: GTGGCACACCGTGAAGT
rs9344 (11q13.3)	56	*CCND1*	exon 4	33 cycles: 95°C 1 min, 57°C 1 min, 72°C 1 min; final extension: 72°C 45 min	177 bp	Fw: CCAACAACTTCCTGTCCTACT
						Rw: CCCAACCTTGTCACCCTT
D9S171 (9p21.3)	71	*CDKN2A/2B*	2.5 Mb downstream	32 cycles: 94°C 1 min, 58°C 1 min, 72°C 50 s; final extension: 72°C 45 min	158–177 bp	Fw: [6FAM]AGCTAAGTGAACCTCATCTCTCTGTCT
						Rw: ACCCTAGCACTGATGGTATAGTCT
D17S796 (17p13.2)	77	*TP53*	1.3 Mb upstream	30 cycles: 94°C 1 min, 58°C 1 min, 72°C 1 min; final extension: 72°C 45 min	144–174 bp	Fw: [HEX]CAATGGAACCAAATGTGGTC
						Rw: AGTCCGATAATGCCAGGATG
D17S578 (17p13.1)	69	*TP53*	0.75 Mb upstream	32 cycles: 94°C 1 min, 58°C 1 min, 72°C 1 min; final extension: 72°C 45 min	134–174 bp	Fw: [6FAM]CTATCAATAAGCATTGGCCT
						Rw: CTGGAGTTGAGACTAGCCT
rs11078663 (17p13.1)	60	*TP53*	0.63 Mb upstream	35 cycles: 94°C 1 min, 58°C 1 min e 30 s, 72°C 1 min; final extension: 72°C 45 min	198 bp	Fw: TGTAGCTCAGGCTCCCA
						Rw: CCATTCCACTTACCTGAGAGAG
D18S363 (18q21.2)	85	*SMAD4*	0.27 Mb upstream	30 cycles: 94°C 1 min, 56°C 1 min, 72°C 1 min; final extension: 72°C 45 min	177–247 bp	Fw: [6FAM]GAAGATTTGGCTCTGTTGA
						Rw: TGTCTTACTGCTATAGCTTTCATAA
D18S474 (18q21.2)	82	*SMAD4*	0.08 Mb downstream	35 cycles: 94°C 1 min, 61°C 1 min, 72°C 1 min; final extension: 72°C 45 min	119–139 bp	Fw: [HEX]TGGGGTGTTTACCAGCATC
						Rw: TGGCTTTCAATGTCAGAAGG

The informativeness of each marker was evaluated in the constitutive DNA, and the analysis was only carried out in the cfDNA of heterozygous individuals. All samples were tested in duplicate to assess data reproducibility. Fragment analysis for the evaluation of LOH events and sequencing analysis for the detection of allelic imbalance were carried out by capillary electrophoresis using the 3730XL DNA analyzer (Life Technologies, Monza, Italy). LOH was calculated by dividing the allele ratio in cfDNA by the allele ratio in germline DNA. Considering the different tumor DNA concentration, LOH positivity was set at ≥35% reduction in one allele for cfDNA and at ≥40% for FFPE-DNA samples. Since Sanger sequencing is not quantitative, the SNP imbalance was arbitrarily defined as a reduction in the peak of at least 50% compared to the germline reference DNA. The global alterations index was calculated by dividing the number of positive loci by the number of informative loci and was defined as Fractional Alteration (FA) index.

### Statistics

Categorical and continuous data were analyzed using Fisher's exact test and Mann-Whitney *U* test, respectively. Pearson's correlation test was applied to calculate the correlation coefficient between cfDNA and FFPE-DNA. All statistical tests were two-sided; a *P*-value < 0.05 was considered significant.

## Results

### Clinicopathological Characteristics of Patients

In this study, we analyzed the cfDNA of 52 patients with EC. The cohort included 33 EADC and 19 ESCC patients, 48 males and 4 females, and the median age at diagnosis was 66 years (Table 2). The fraction of tumor DNA present in the total cfDNA is generally considered proportional to tumor burden (22). Thus, to have more favorable conditions to detect cell free tumor DNA, we enrolled only patients with a T3–T4 neoplasia at diagnosis or at restaging after neoadjuvant therapy. The majority of patients (83%) had at least one positive lymph node (Table 2). All patients had surgical resection after neoadjuvant chemo-radiotherapy; only one patient underwent surgery directly due to frailty. Four patients were also analyzed longitudinally.

**Table 2:** Clinicopathological characteristics of EC patients.

**Patients**	**Total**
	***N* (%)**
	**52 (100)**
**Age**	
Median (IQR)	66 (57–73)
(Range)	(42–79)
**Gender**	
Male	48 (92)
Female	4 (8)
**Histotype**	
EADC	33 (63)
ESCC	19 (37)
**TNM**	
**T**	
3	51 (98)
4	1 (2)
**N**	
0	9 (17)
1+	43 (83)
**M**	
0	51 (98)
1	1 (2)

*IQR, interquartile range; EADC, Esophageal adenocarcinoma; ESCC, Esophageal squamous cell carcinoma*.

### Genetic Alterations Analysis in cfDNA

**Figure 1:**
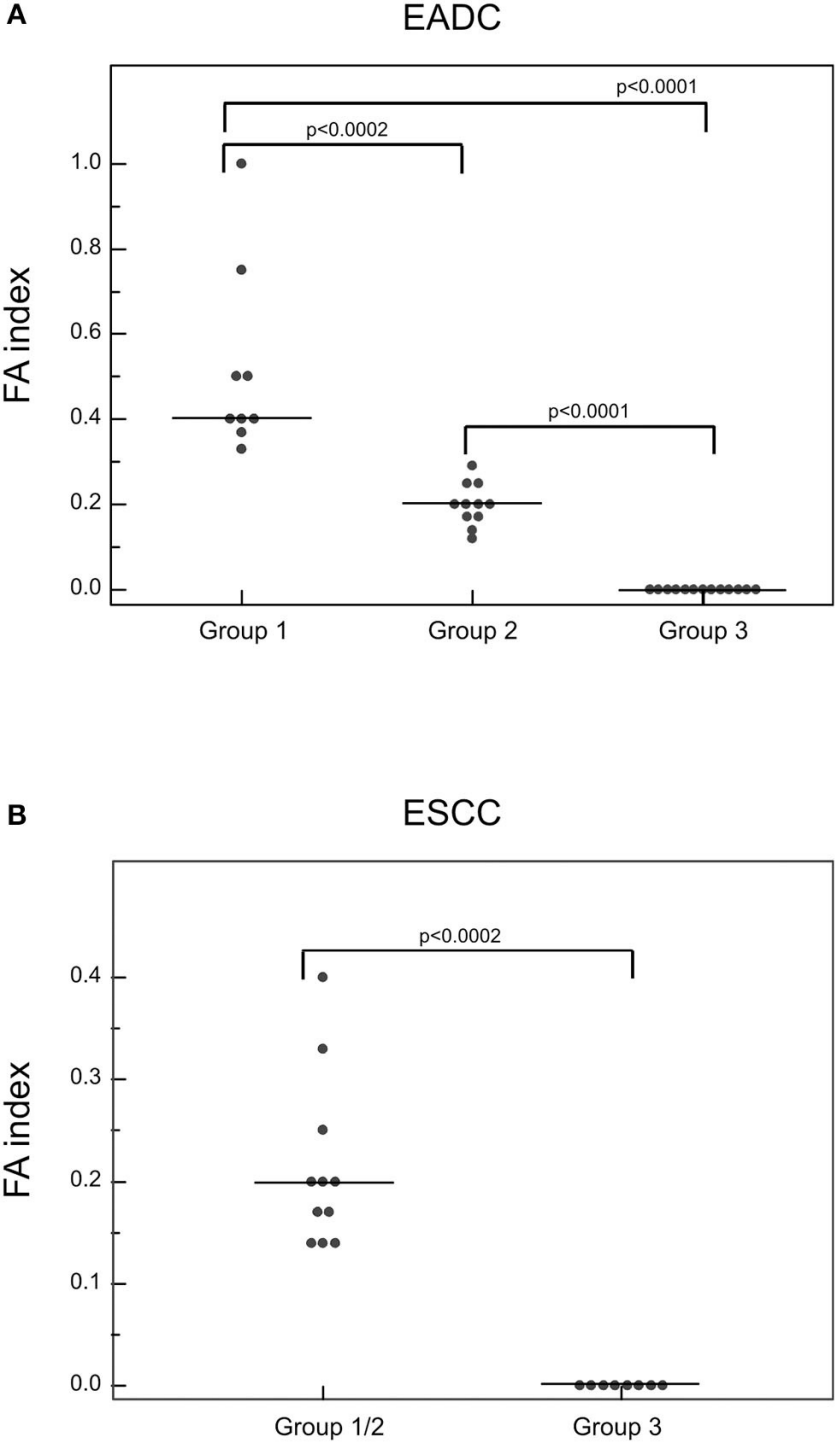
Detection of genetic alterations in cfDNA of EADC **(A)** and ESCC **(B)** patients. The plotted values correspond to the FA index obtained from dividing the number of positive markers by the total number of informative loci. *P-*values were calculated using the Mann-Whitney *U* test. Group 1 represents the highly altered profile group; group 2, the intermediate altered profile group; and group 3, the not altered profile group.

**Figure 2:**
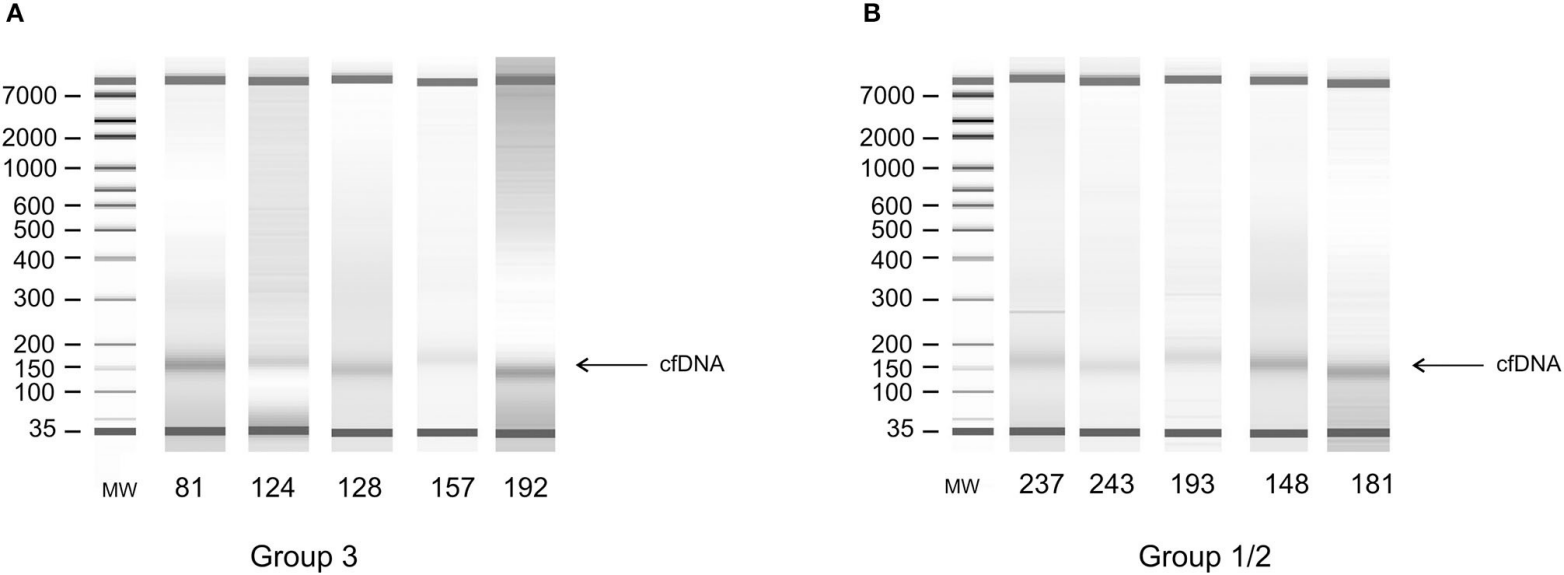
Electrophoretic runs of DNA extracted from plasma. **(A)** Random selected samples from alteration-negative (group 3) cfDNA. **(B)** Random selected samples from alteration-positive (group 1 or 2) cfDNA.

All cfDNA samples were tested for the presence of tumor-related genetic alterations using as markers a custom panel of 5 MSs and 3 SNPs. The chosen markers map within or near suppressor genes or oncogenes reported to be altered or lost with a high frequency in EC (3, 5). Results showed that, according to our markers and to their fractional alteration (FA) index, EADC patients could be stratified into three subgroups (Figure 1A). Group 1 showed a highly altered profile (median FA index 0.40, range 1–0.30), group 2 had an intermediate profile (median FA index 0.20, range 0.29–0.12), while group 3 was characterized by the absence of any alterations in the considered markers (FA index 0). ESCC patients could also be divided into subgroups: one (group 1/2) with a high/intermediate number of alterations (median FA index 0.20, range 0.40–0.14) and another (group 3) with no detectable alterations (FA index 0) (Figure 1B). In both histotypes the FA index of each subgroup was statistically different (*P* value ranging from <0.0002 to <0.0001). In order to verify whether the absence of any detectable alteration was due to a low quantity or a bad quality of cfDNA, we performed capillary electrophoretic runs of a few randomly selected marker-negative (group 3) samples; as a control, cfDNA of a few randomly chosen marker-positive (group 1 or 2) samples were included in the runs. The resulting quality and quantity of cfDNA were quite similar between the two groups (Figure 2), indicating that the non-detectability of the selected tumor-related alterations was not ascribable to technical problems.

When we considered the alterations in their totality, we found that EADC and ESCC had a comparable number of alterations (median FA index: 0.17 vs. 0.14). The locus of tumor suppressor *TP53* was the most altered in both EC histotypes (48% in EADC vs. 47% in ESCC; Table 3), although MS D17S796, the most distal to *TP53*, was not altered in ESCC. Similar frequencies of imbalance were also observed for the markers mapping *TP63* (12 vs. 10%) and *CCND1* (19 vs. 13%), while losses at the *CDKN2A/B* locus were higher in EADCs (25 vs. 15%), although not statistically relevant (*P* = 0.68). On the contrary, LOH at the *SMAD4* locus was a peculiarity of EADCs. Indeed, considering D18S363 and D18S474, located, respectively, upstream and downstream of *SMAD4*, we found a LOH frequency of 26% in EADC vs. 0% in ESCC samples (*P* = 0.018) (Table 3).

**Table 3:** Alteration frequencies at single marker or locus level.

		**Single marker**			Locus^*^		
**Marker ID**	**Involved gene**	**EADC**	**ESCC**	***p*-value**	**EADC**	**ESCC**	***p*-value**
rs28673064	*TP63*	12%	10%	1			
rs9344	*CCND1*	19%	13%	1			
D17S796	*TP53*	12%	0%	0.28	48%	47%	1
D17S578		41%	28%	0.50			
rs11078663		22%	38%	0.43			
D9S171	*CDKN2A*	25%	15%	0.68			
D18S363	*SMAD4*	15%	0%	0.13	26%	0%	0.018
D18S474		19%	0%	0.14			

* *When determining alterations at a locus mapped by more than one marker, an alteration was considered present if it appeared in at least one of the markers; only one alteration per locus has been counted*.

### Analysis of Concordance Between cfDNA and Tumor DNA

**Figure 3:**
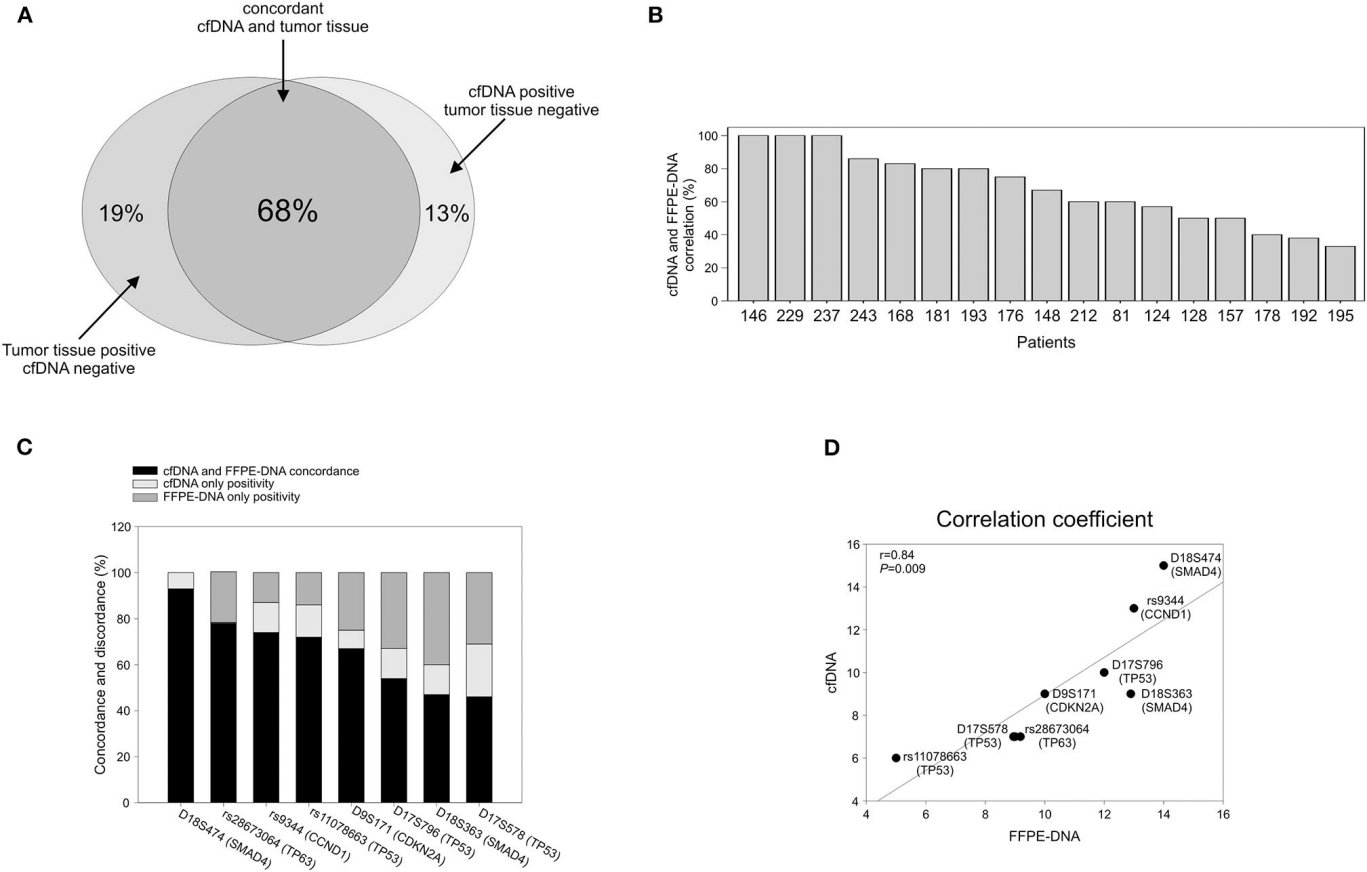
Correlation between cfDNA and time-matched tumor DNA. **(A)** Global concordance. **(B)** Concordance at the single patient level. **(C)** Concordance at the single marker level. **(D)** Graphic representation of the Pearson's correlation coefficient.

To verify whether the alterations detected in cfDNA were representative of those present in tumor tissue, we analyzed tumor DNA isolated from 17 time-matched EADC FFPE specimens. Considering all the markers together, we found that the average of the overall concordance between tumor DNA and cfDNA was 68% with a 32% discrepancy divided into 19% positivity only in tumor DNA and 13% only in cfDNA (Figure 3A). The concordance was quite variable from individual to individual with three patients having a 100% concordance; four, a concordance ≥80%; five, a concordance >50%; and the remaining five patients, a concordance ranging from 50 to 33% (Figure 3B). When we estimated the concordance at the level of individual markers, we observed that, among the eight analyzed markers, some had a better match than others. As reported in Figure 3C, rs28673064 (*TP63*), rs9344 (*CCND1*), and rs11078663 (*TP53*) exhibited an individual concordance >70%. MS D18S474 (*SMAD4*) was even better, showing a concordance of 93% with a few discrepant samples being positive only in cfDNA. This last result might reflect the capability of cfDNA to better represent tumor heterogeneity rather than a false outcome. Altogether, the eight markers exhibited a 0.84 correlation coefficient with a significance of *P* = 0.009 (Figure 3D).

### Analysis of Longitudinal Cases

cfDNA analysis, among its many potentialities, has been indicated as a possible method for tumor detection in patients without clinical evidence of the disease (15, 16). Thus, we studied a few EC patients longitudinally to see whether the search for tumor markers in cfDNA could be useful for monitoring the patients during their therapeutic journey. Here, we reported data regarding four representative patients.

**Figure 4:**
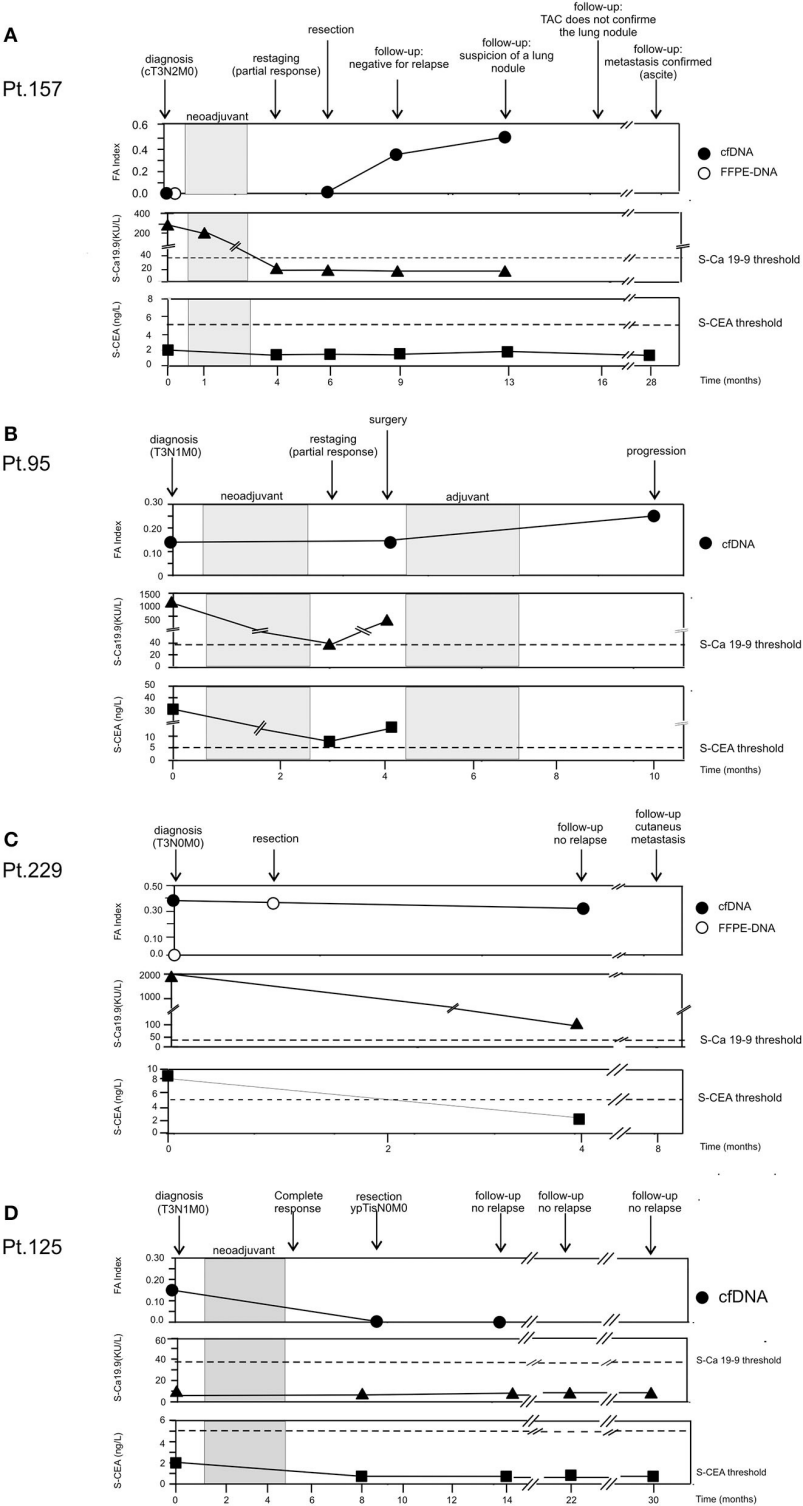
Longitudinal analysis. cfDNA was isolated from serial plasma. CEA and Ca 19.9 values were obtained from patient's clinical record. **(A)** Patient 157. **(B)** Patient 95. **(C)** Patient 229. **(D)** Patient 125.

**Patient 157**. Figure 4A The patient presented an adenocarcinoma (cT3N2M0) at diagnosis. Both cfDNA and FFPE-DNA resulted negative for our genetic markers as well as the cfDNA obtained at surgery. However, 3 months after resection, although no clinical signs of recurrence were present and the tumor soluble markers S-CEA and S-Ca 19.9 remained below the threshold of positivity, the FA index became highly positive (0.33). At the next follow-up, 3 months later, the FA index increased further (0.50); at this time, a suspicion of a lung nodule was advanced. However, the lump was not confirmed at radiological examination 3 months later; soluble tumor markers continued to be negative. One year later, the patient presented pleural effusion and the lung metastasis was confirmed. S-CEA and S-Ca 19.9 were still negative.

**Patient 95**. Figure 4B Blood samples were collected at the diagnosis of adenocarcinoma (T3N1M0) at restaging after neoadjuvant treatment and at 6 months after surgery. At the time of diagnosis, it was possible to detect tumor-related alterations in the cfDNA sample (FA index: 0.17), and the tumor soluble markers S-CEA and S-Ca 19.9 were highly positive. After neoadjuvant therapy, S-CEA and S-Ca 19.9 dropped but remained above the threshold of positivity; on the contrary, FA index did not change. Surgical resection was not curative and a cycle of adjuvant therapy was scheduled. The tumor did not respond to treatment and progressed; FA index doubled and reached the value of 0.33.

**Patient 229**. Figure 4C At the diagnosis of adenocarcinoma (T3N0M0), genetic alterations were found in cfDNA (FA index 0.37), but not in the time-matched FFPE-DNA; the patient was also positive for soluble tumor markers. Because of the poor state of general health, the patient underwent surgery without neoadjuvant therapy. Interestingly, the DNA isolated from the FFPE surgical specimen resulted positive for alterations with a 75% concordance with the alterations found in cfDNA collected at diagnosis. At follow-up performed 3 months after surgery, the patient did not present clinical signs of relapse; S-CEA became negative; and S-Ca 19.9, although still positive, was far below the initial value. On the contrary, the FA index remained high after tumor resection. Four months later, the patient presented cutaneous metastasis.

**Patient 125**. Figure 4D At diagnosis of squamous cell carcinoma (T3N1M0), the patient was negative for the tumor soluble markers S-CEA and S-Ca 19.9. By contrast, genetic alterations were detected in cfDNA (FA index 0.17). The patient had a complete response to neoadjuvant therapy (ypTisN0M0). At surgery, cfDNA resulted negative for the presence of tumor-related alterations and remained negative 5 months later. The patient did not show clinical signs of relapse during a 30-month follow-up.

## Discussion

EC is a highly aggressive tumor of which the survival rate remains low despite the application of multimodal therapeutic protocols (23). In addition, current monitoring procedures sometimes do not adequately account for the efficacy of treatments or the risk of relapse. For this reason, we investigated whether liquid biopsy could be used, alongside current methods, for the monitoring of locally advanced EC patients.

ECs are characterized by high mutational frequencies and recurrent losses/gains of tumor suppressor genes or oncogenes. Using a panel of 5 MSs and 3 SNPs that map near loci highly altered in EC (3, 5, 21), we were able to find tumor-related alterations in cfDNA of EC patients and follow the disease longitudinally.

No statistical differences were observed between EADC and ESCC when the alterations were globally considered. Also, at the level of a single genetic marker, the frequency of alterations was quite similar, and in line with the genetic alteration landscape of EC, we observed a high proportion of deletions at the *TP53* locus in both histotypes. Interestingly, ESCCs did not have LOH at the MS most distal to *TP53* (D17S796), suggesting that the LOH event at the *TP53* locus is probably wider in EADC.

In line with the literature data (3, 24, 25), EADC exhibited loss of the *SMAD4* locus with a good frequency; this event was peculiar to EADC since ESCC did not exhibit any loss at this locus (*P* = 0.018). In addition, the frequency of *TP63* and *CCND1* loci in EADC were similar to previous reported data (12 vs. 11% for *TP63* and 19 vs. 15% for *CCND1*) (3). On the contrary, the frequency of these loci was lower than expected in ESCC samples. This discrepancy might be due to the small number of informative samples for these markers in ESCC cohort.

Moreover, not all the studied patients exhibited alterations in at least one of the eight chosen markers. Indeed, almost 1/3 did not show any change, although the quality and quantity of their cfDNA were comparable to those of positive samples. These data are in line with the 64–70% mutation positivity in driver genes found using next-generation sequencing (NGS) in larger cohorts of lung cancer patients (26, 27). In our cohort, this negativity was also observed in a few time-matched tumor DNA (i.e., Pt. 157; Figure 4A and data not shown), suggesting that these samples are most likely characterized by other genetic events. Despite the limited number of analyzed cases, these findings suggest that both EADC and ESCC can be stratified into subgroups that differ in the number and, perhaps, in the type of alterations. No correlation between the FA index and tumor progression was observed, indicating that alterations at the analyzed markers or their number are not linked to a more aggressive disease. Nonetheless, the recognition of subgroups that differ for the number of molecular alterations could be relevant for therapy stratification of EC patients. Indeed, patients with a high FA index could be putatively eligible for an immunotherapeutic approach.

When we compared the alterations detectable in cfDNA and those present in the time-matched tumor DNA, we found a global correlation of 68% with, among the discordant samples, 13% alteration-positive only in the cfDNA. This finding could be ascribable to the hypothesized greater representativeness of cfDNA of tumor heterogeneity with respect to a single tissue biopsy (16, 28, 29). This hypothesis is also sustained by the results obtained in Pt. 229 (Figure 4C). Indeed, while the cfDNA obtained at diagnosis and the matched tissue biopsy-DNA were discordant (i.e., alterations vs. no alterations, respectively), the tumor DNA obtained from the specimen at surgery had 75% concordance with the cfDNA gathered at diagnosis.

Data from longitudinal cases indicate that cfDNA analysis can be useful to follow EC patient response to neoadjuvant treatment or to determine whether surgical resection was curative. In some cases, the resulting FA index was more reliable than traditional soluble tumor markers such as S-CEA and S-Ca 19.9 to indicate tumor progression. Indeed, tumors that were also positive for S-CEA or S-Ca 19.9 at diagnosis sometimes did not retain their positivity during progression. On the contrary, FA index never became negative during progression.

The limitations of this study are its retrospective nature and the relatively low number of patients enrolled, which renders it an exploratory and hypothesis-driven study that needs further prospective confirmatory trials. Nevertheless, our work is in line with previous studies that, using next-generation sequencing technology (NGS), highlighted the relevance of cfDNA analysis to follow EC patient behavior (30–32). The Kato et al. (30) and Maron et al. (32) studies have cohorts that include mainly gastric and junction adenocarcinomas. More similar to our study is the paper of Azad et al. (31), which includes a cohort of 45 EADC and ESCC patients. This data concordance highlights our findings and technical approach.

Thus, despite its limitations, our study indicates the validity of our approach that is easy to perform and economically sustainable. Furthermore, we confirmed the capacity of liquid biopsy to assess response to neoadjuvant therapy and to detect a putative residual disease before instrumental examination, as suggested by longitudinal studies. Although further confirmatory studies are required, we believe that in the near future, liquid biopsy could be used alongside the current EC patient monitoring strategies to guide and improve patient management.

## Data Availability Statement

The raw data will be made available upon motivated request.

## Ethics Statement

The studies involving human participants were reviewed and approved by Comitato Etico per la Sperimentazione Clinica (CESC) of the Veneto Institute of Oncology. The patients/participants provided their written informed consent to participate in this study.

## Author Contributions

EB, MC, and DS: conception and design, analysis and data collection, interpretation of data, and manuscript preparation. MF, MR, SR, and RA: sample collection. EB, MC, DS, MF, and AA: critical revision of manuscript. AA: funding acquisition. All authors contributed to the article and approved the submitted version.

## Conflict of Interest

The authors declare that the research was conducted in the absence of any commercial or financial relationships that could be construed as a potential conflict of interest.
